# Impact of *ABCG2* polymorphisms on the clinical outcome of TKIs therapy in Chinese advanced non-small-cell lung cancer patients

**DOI:** 10.1186/s12935-015-0191-3

**Published:** 2015-04-19

**Authors:** Xueqin Chen, Dadong Chen, Shaoyu Yang, Ruobing Ma, Yuelong Pan, Xin Li, Shenglin Ma

**Affiliations:** Department of Medical Oncology, Nanjing Medical University, Affiliated Hangzhou Hospital(Hangzhou First People’s Hospital), 261 Huansha Road, Hangzhou, Zhejiang Province 310006 China

**Keywords:** ATP binding cassette superfamily G member 2, Polymorphism, Non-small-cell lung cancer, Overall survival, Tyrosine kinase inhibitions (TKIs) therapy

## Abstract

**Objective:**

The primary purpose of this study was to investigate the correlation between single nucleotide polymorphisms (SNPs) of ATP binding cassette superfamily G member 2 (*ABCG2*) and outcome of tyrosine kinase inhibitions (TKIs) therapy in Chinese advanced non-small-cell lung cancer (NSCLC) patients. The secondary objective was to identify biomarkers to evaluate the response to treatment and outcome of the targeted therapy.

**Methods:**

SNP genotyping (34 G/A, 421 C/A, 1143 C/T and −15622 C/T) of *ABCG2* gene in 100 patients was performed using matrix-assisted laser desorption/ionization time-of-flight mass spectrometry. The clinical characteristics of 100 patients were collected. A total of 70 patients were treated with TKIs (gefitinib, erlotinib and icotinib). The association between *ABCG2* polymorphisms and clinical characteristics was evaluated. Kaplan-Meier survival curves were plotted for overall survival (OS) and analyzed with the log-rank test.

**Results:**

The three polymorphisms of the *ABCG2* 34 G/A, 421 C/A and 1143 C/T occurred more frequently compared with −15622 C/T in Chinese advanced NSCLC patients. There was no association between *ABCG2* polymorphisms and clinical characteristics (*p* > 0.05). The median OS of patients with GG genotype at position 34 of the *ABCG2* gene was significantly shorter than those with GA or AA genotype (*p* < 0.05). No significant difference of OS was found in 421 C/A and 1143 C/T polymorphisms (*p* > 0.05).

**Conclusion:**

*ABCG2* 34 G/A may be a possible predictor of the clinical outcome of TKIs therapy in NSCLC patients.

**Electronic supplementary material:**

The online version of this article (doi:10.1186/s12935-015-0191-3) contains supplementary material, which is available to authorized users.

## Background

Lung cancer is one of the most prevalent and fatal malignant neoplasm all over the world and non-small-cell lung cancer (NSCLC) accounts for 80%–85% of all lung cancer patients [1]. Approximately 80% of NSCLC patients are diagnosed as advanced (phase IIIA/B) or metastatic (phase IV) stages of the disease [2], which results in quite low 5-year survival rates, 8–14.1% for phase IIIA and 1-5% for phase IIIB/IV [3]. Chemotherapy as the standard treatment of advanced NSCLC has reached a bottleneck with limited effects such as high relapse rates and toxicity [4]. In recent years, targeted therapy has been widely applied in clinical practice to replace chemotherapy. Tyrosine kinase inhibitors (TKIs), targeted drugs of epidermal growth factor receptor (EGFR), have been recently introduced for the treatment of NSCLC [5]. Clinical trials indicated that gefitinib, erlotinib and icotinib, as EGFR-TKIs, are active and valid treatment for patients with advanced or metastatic NSCLC [6-8]. It has been reported that the patients with advanced disease widely received targeted therapy [9]. Therefore, it is essential to find biomarkers to predict the response to treatment and outcome of the targeted therapy.

ATP binding cassette superfamily G member 2 (*ABCG2*) is a member of the ATP-binding cassette (ABC) superfamily of multidrug transporters, which has been involved in tumor cell resistance to anticancer therapy [10]. The *ABCG2* protein is highly expressed in the gastrointestinal tract and blood–brain barrier, where it is thought to play a role in protection against xenobiotic exposure [11]. More than 80 single nucleotide polymorphisms (SNPs) have been found in the *ABCG2* gene [12]. The SNPs in *ABCG2* are indicated to affect the expression of *ABCG2* protein. *ABCG2* protein expression is related to response of advanced NSCLC patients treated with chemotherapy/targeted therapy [13-15]. *ABCG2* 421 C/A polymorphism is strongly associated with gefitinib-induced diarrhea in Caucasian NSCLC patients [14]. Therefore, SNPs in the *ABCG2* gene may influence the pharmacological effects. Besides, SNPs in *ABCG2* gene in Asian population are different from other ethnicities [16], However, the genetic polymorphisms of *ABCG2* gene and its impact on the outcome of targeted therapy in Chinese advanced NSCLC patients are still not clearly demonstrated.

In this study, we tested the polymorphism of *ABCG2* 34 G/A (rs2231137), 421 C/A (rs2231142), 1143 C/T (rs2622604) and −15622 C/T (rs7699188) in 100 Chinese advanced NSCLC patients and analyzed the association of SNPs in *ABCG2* gene with clinical characteristics and clinical outcome for NSCLC patients treated with TKIs therapy. We expect the study can supply insights to validate the role of *ABCG2* polymorphisms for elective treatment and to improve patients’ quality of life.

## Materials and methods

### Patients and treatment

A total of 100 patients with ECOG performance status of 0 to 2, and pathology and cytology confirmed advanced or metastatic NSCLC were enrolled into this study between April 2012 and January 2014 in Hangzhou, China. TKIs targeted therapy was implemented in 70 NSCLC patients and the other therapy was implemented in the remaining patients. In this study, clinical outcome was only measured in the TKIs targeted therapy, not others. Patients subjected to TKIs targeted therapy were treated with gefitinib (Astrazeneca pharmaceutical co., LTD, Wuxi, China) at a dose of 250 mg once a day or erlotinib (Roche pharmaceuticals co., LTD, Shanghai, China) at a dose of 150 mg once daily or icotinib (Zhejiang beida pharmaceutical co., LTD, Hangzhou, China) at a dose of 125 mg three times a day until disease progression or intolerable toxicity. All patients received chest CT every 2 months after 1 month of therapy.

The efficacy of TKIs therapy was clarified as complete response (CR), partial response (PR), stable disease (SD) and progression disease (PD) according to the Response Evaluation Criteria In Solid Tumors (RECIST) 1.1 [17]. Patients of CR, PR and SD for more than 6 months were considered as sensitive to treatment. Patients of SD for less than 6 months and PD were considered as resistant to treatment. Progression-free survival (PFS) was defined as the duration in month from start of TKI therapy to disease progression. Overall survival (OS) was calculated from the time of diagnosis until death from any cause.

All patients agreed to participate in this study and signed written informed consent. This study was approved by the Institutional Review Board of Nanjing Medical University Affiliated Hangzhou Hospital and performed in accordance with the Declaration of Helsinki and Good Clinical Practice guidelines.

### DNA extraction

Blood samples were collected before TKIs therapy and kept in a microcentrifuge tube containing ethylenediamine tetra-acetic acid (EDTA). Genomic DNA was extracted from whole blood using a DNA purification kit (Flexi Gene DNA Kit, Qiagen, Hilden, Germany). The concentration of genomic DNA was determined with NanoDrop 1000 (Thermo Scientific, Wilmington, USA) and then diluted to a standard of 25 ng/μl.

### Analysis of *ABCG2* polymorphisms

The *ABCG2* 34 G/A was amplified using the primers *ABCG2* 34 G/A Forward (5′-ACGTTGGATGTCAGGTCATTGGAAGCTGTC-3′), Reverse (3′-ACGTTGGATGGATGTCTTCCAGTAATGTCG-5′), and UEP_SEQ which means the primer for single base extension reaction (GTGTCGAAGTTTTTATCCCA). The *ABCG2* 421 C/A was amplified using the primers *ABCG2* 421C/A Forward (5′-ACGTTGGATGTGATGTTGTGATGGGCACTC-3′), Reverse (3′-ACGTTGGATGGTCATAGTTGTTGCAAGCCG-5′), and UEP_SEQ (AGAGCTGCTGAGAACT). The *ABCG2* 1143 C/T was amplified using the primers 1143 C/T Forward (5′-ACGTTGGATGACTCTGAAAGCACTGTTTTG-3′), Reverse (3′-ACGTTGGATGCATTTGAATGTCAGCTAGTC-5′), and UEP_SEQ (TGTCAGCTAGTCATAAATAAATAC). Moreover, The *ABCG2* -15622 C/T was amplified using the primers ABCG2 -15622C/T Forward (5′-ACGTTGGATGCTGGCCAAGACCCTATCTTA-3′), Reverse (5′- AACGTTGGATGGCCACCTATCTTTGTTCACC-3′) and UEP_SEQ (TTAGGACTACAGACATGC).

Finally, genotyping of *ABCG2* SNPs were conducted using matrix-assisted laser desorption/ionization time-of-flight mass spectrometry (MALDI-TOF MS) and Sequenom MassARRAY system (Sequenom, San Diego, CA, USA).

### Statistical analysis

Allele frequencies of SNPs were calculated and their genotype distributions were assessed by Hardy-Weinberg Equilibrium using the chi-square test.

PFS and OS with 95% confidence intervals (CI) were evaluated with censored survival time methods. Kaplan-Meier survival curves were plotted for OS and analyzed with the log-rank test.

The univariate analysis included several clinical characteristics. Baseline characteristics included gender, age (≤63 vs >63), histology (adenocarcinoma vs others), smoking (never vs ever). A multivariate logistic regression model was used to analyze the clinical outcomes in NSCLC patients treated by TKIs and estimate the adjusted hazard ratio (HR) and its 95% CI. Cox proportional hazards model was applied to evaluate the association between OS and clinical or genomic characteristics and estimate the adjusted HR and its 95% CI.

All tests were 2-sided and a *p*-value < 0.05 was considered statistically significant. All statistical analyses were carried out using SPSS 18.0 (SPSS Inc., Chicago, IL, USA) software.

## Results

### Characteristics of the study patients

The detailed characteristics of patients were given in Table 1. There were 53 male and 47 female patients enrolled for study. The average age was 64 years. Sixty-six patients had history of cigarette smoking. There were 81 cases of adenocarcinomas in pathological classification of primary tumors. Patients with EGFR gene mutation accounted for 21% of total cases. Totally, 39, 10 and 21 patients separately received gefitinib, erlotinib or icotinib in our study.

**Table 1:** **Main clinical characteristics for the non-small cell lung cancer (NSCLC) patients**

	**N = 100**	**%**
**Gender**		
Male	53	53%
Female	47	47%
**Age**		
Median (range)	64	36-83
≤64	54	54%
>64	46	46%
**Smoking history**		
Never	66	66%
Ever	34	34%
**Histological**		
Adenocacinoma	81	81%
Others	19	19%
**EGFR mutation**		
Positive	21	21%
Negative	11	11%
Unknown	68	68%
**Patients treated with TKI**		
Gefitinib	39	39%
Erlotinib	10	10%
Icotinib	21	21%

### *ABCG2* gene polymorphisms

The genotyping of *ABCG2* 34 G/A, 421 C/A, 1143 C/T and −15622 C/T were performed in all these 100 patients. Moreover, the genotyping and allele frequencies of *ABCG2* in a Chinese NSCLC population were shown in Table 2. Regarding the *ABCG2* -15622 C/T polymorphism, the TT genotype was observed in all patients. Therefore, polymorphism of *ABCG2* -15622 C/T was not investigated in the following steps.

**Table 2:** **Genotyping and allele frequencies of** ***ABCG2*** **in a Chinese NSCLC population**

**SNP**	**Genotype**	**Number**	**Frequencies**	**Allele**	**Frequencies**
**34 G/A**	GG	36	0.36	G	0.610
	GA	50	0.50	A	0.390
	AA	14	0.14		
**421 C/A**	CC	53	0.53	C	0.745
	CA	43	0.43	A	0.255
	AA	4	0.40		
**1143 C/T**	CC	66	0.66	C	0.805
	CT	29	0.29	T	0.195
	TT	5	0.50		
**−15622 C/T**	CC	0	0	C	0
	CT	0	0	T	1.000
	TT	100	1.00		

### Association between polymorphisms of *ABCG2* and clinical characteristics

The association between polymorphisms of *ABCG2* and clinical characteristics were presented in Table 3. No significant correlation was found between *ABCG2* polymorphisms (34 G/A, 421 C/A and 1143 C/T) and clinical characteristics, including gender, age, smoking history, histology and EGFR mutation (*p* > 0.05).

**Table 3:** **The association between** ***ABCG2*** **polymorphisms and clinical characteristics**

	**34 G/A**			***P*** **-value**	**421 C/A**			***P*** **-value**	**1143 C/T**			***P*** **-value**
	**GG**	**AG**	**AA**		**CC**	**CA**	**AA**		**CC**	**TC**	**TT**	
**Gender**				0.684				0.501				0.682
Male	19	28	6		31	20	2		34	17	2	
Female	17	22	8		22	23	2		32	12	3	
**Age**				0.725				0.167				0.517
≤64	18	29	7		28	22	4		34	18	2	
>64	18	21	7		25	21	0		32	11	3	
**Smoking history**				0.564				0.667				0.527
Never	23	32	11		34	30	2		45	17	4	
Ever	13	18	3		19	13	2		21	12	1	
**Histology**				0.955				0.816				0.535
Adenocacino	29	41	11		42	36	3		53	23	5	
Others	7	9	3		11	7	1		13	6	0	
**EGFR mutation**				0.204				0.119				0.305
Positive	7	14	0		9	10	2		15	6	0	
Negative	3	6	2		9	2	0		6	4	1	

### Association between polymorphisms of *ABCG2* and clinical outcome of TKIs therapy

The sensitivity of 70 patients subjected to TKI treatment was presented in Table 4. None of the three SNPs (34 G/A, *p* = 0.453; 421 C/A, *p* = 0.615 and 1143 C/T, *p* = 0.804) was significantly correlated with sensitivity.

**Table 4:** **Association between** ***ABCG2*** **polymorphism and clinical outcome of tyrosine kinase inhibitions (TKIs) therapy in 70 NSCLC patients**

	**Clinical outcome**		***P*** **-value**	**PFS (95% CI)**	***P*** **-value**	**OS (95% CI)**	***P*** **-value**
	**Sensitive (n)**	**Resistive (n)**					
**34 G/A**							
GG	15	10		6.5 (4.1-8.9)		18 (14.9-21.1)	
GA + AA	31	14	0.453	8.0 (5.9-10.1)	0.355	31 (22.9-39.1)	0.005
**421 C/A**							
CC	22	13		7.0 (3.7-10.4)		19 (7.4-30.6)	
CA + AA	24	11	0.615	8.0 (6.1-9.9)	0.089	28 (15.0-41.0)	0.823
**1143 C/T**							
CC	32	16		8.0 (5.4-10.7)		21 (11.0-30.0)	
CT + TT	14	8	0.804	6.5 (3.12-9.8)	0.082	27.5 (15.4-39.6)	0.872

PFS: progression-free survival; OS: overall survival; 95% CI: 95% confidence intervals.

Median PFS for carriers of the A-allele and GG genotype at position 34 of the *ABCG2* gene who were treated with TKIs therapy was 8.0 months (95% CI: 5.9-10.1, n = 45) and 6.5 months (95% CI: 4.1-8.9, n = 25), respectively. However, no significant difference was found (*p* = 0.355). Similarly, there was no significant difference in median PFS of NSCLC patients receiving TKIs therapy between CC genotype and CA/AA genotype at position 421 of *ABCG2* gene (*p* = 0.089). Median PFS of patients with CC genotype at position 1143 of *ABCG2* gene was higher than those with CT or TT genotype, but no significant difference was found (*p* = 0.082).

**Figure 1:**
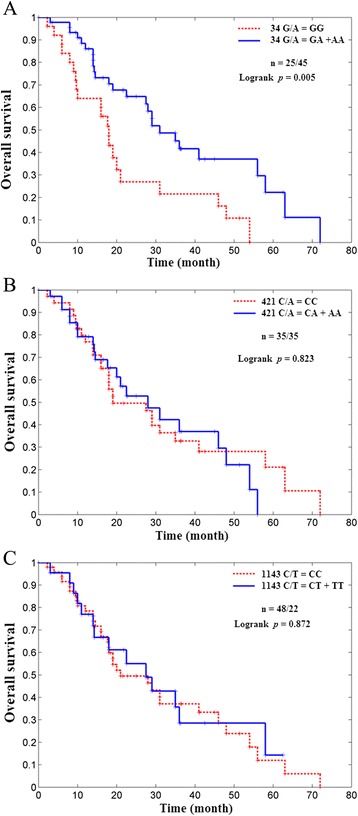
Kaplan–Meier curve shows overall survival (OS) for the non-small-cell lung cancer (NSCLC) patients receiving tyrosine kinase inhibitors drugs related to ATP binding cassette superfamily G member 2 (*ABCG2*) genotypes at 34 G/A (**A**, *p* = 0.005), 421 C/A (**B**, *p* = 0.823) and 1143 C/T (**C**, *p* = 0.872).

The median OS of patients with GG genotype at position 34 of the *ABCG2* gene was 18 months (95% CI: 14.9-21.1, n = 25) while the median OS for those with other genotypes (GA or AA) was 31 months (95% CI: 22.9-39.1, n = 45). Figure 1 showed the Kaplan-Meier curve for OS for NSCLC patients receiving TKIs therapy in relation to *ABCG2* genotypes at 34 G/A (Figure 1A), 421 C/A (Figure 1B) and 1143 C/T (Figure 1C). There was significant difference between patients with GG genotype and those with GA or AA genotype at position 34 of the *ABCG2* gene (*p* = 0.005). However, there was no significant difference between patients with CC genotype regarding the position 421 of *ABCG2* gene and carriers with other genotypes (CA or AA, *p* = 0.823). Similarly, no significant difference was found in 1143 C/T polymorphism (*p* = 0.872).

Univariate analysis exhibited that gender (HR 1.615; 95% CI 0.922-2.955; *p* = 0.092), age (HR 1.371; 95% CI 0.761-2.472; *p* = 0.294), smoking (HR 1.477; 95% CI 0.785-2.779; *p* = 0.227) and histology (HR 2.213; 95% CI 0.681-7.194; *p* = 0.187) had no significant effect on OS. Similarly, *ABCG2* C421A as well as C1143T polymorphism also had no significant influence on the OS. On the contrary, *ABCG2* G34A was a statistically significant factor for the OS endpoints in all patients (HR 1.526; 95% CI 1.128-2.065; *p* = 0.006). Gender had no significant influence. The results of univariate analyses were shown in Additional file 1: Table S1.

Similarly, the OS was independently associated with *ABCG2* G34A (HR 1.765; 95% CI 1.193-2.611; *p* = 0.004) based on multivariate analysis of gender, age, smoking, histology, and the *ABCG2* C421A as well as C1143T polymorphism (Additional file 1: Table S1).

## Discussion

In the present study, we detected the polymorphism of *ABCG2* 34 G/A, 421 C/A, 1143 C/T and −15622 C/T in 100 Chinese advanced NSCLC patients. The associations between *ABCG2* polymorphisms and clinical characteristics and clinical outcome for patients treated with TKIs therapy were analyzed. The results showed that three polymorphisms (34 G/A, 421 C/A and 1143 C/T) of the *ABCG2* gene occurred more frequently compared with −15622 C/T in Chinese advanced NSCLC patients. No significant correlations were found between *ABCG2* polymorphisms (34 G/A, 421 C/A and 1143 C/T) and clinical characteristics. The median OS of patients with GG genotype at position 34 of the *ABCG2* gene was significantly shorter than those with GA/AA genotype.

The polymorphisms of *ABCG2* gene are different in different ethnic groups [16]. The allele frequency of the 34 G/A variant in East Asian populations including Chinese (20.0%), Koreans (19.8%) and Japanese (15.0-19.0%) is very similar [16]. However, it is much lower than that in Southeast Asians (45%) and higher than other ethnic groups including Caucasian (1.7–10.3%), African-American (6.3%) and Middle Eastern (5.0%) populations [16]. Similarly, the frequency of 421 C/A variant is similar to the Asian populations, but very different to the other ethnic groups [16]. In Caucasians, the frequency of 421 C/A variant was reported to be 28% [18]. In this study, the frequencies of *ABCG2* polymorphisms 34 G/A, 421 C/A and 1143 C/T were higher than −15622 C/T, which was consistent with a previous study reported by Kobayashi *et al*. [19] suggestting that the most frequent *ABCG2* polymorphisms were 34 G/A and 421 C/A. Interestingly, all patients in this study were observed TT genotype at −15622 C/T position. As far as we know, this gene has not been investigated in other Asian populations. Therefore, further studies could be conducted to determine the polymorphism of −15622 C/T in Asian population and its potential impact.

The polymorphisms of *ABCG2* gene are associated with the protein expression or function in NSCLC [20]. High *ABCG2* expression has been correlated with multidrug resistance and poorer clinical outcomes, as drug transporter has the ability to extrude its drug substrates out of the cells, thereby decreasing their intracellular accumulation [21,22]. Therefore, *ABCG2* plays an important role in determining drug disposition. According to the Kaplan-Meier curve and Cox regression analysis in our study, *ABCG2* 34 G/A showed a significant positive influence on the OS for carriers of the A allele in NSCLC patients, which led to longer OS than those with wild type (GG). Significantly, our results were in line with the previous studies which reported that *ABCG2* 34 G/A polymorphisms were associated with the occurrence of grade 2 of worse skin rash in NSCLC patients treated by gefitinib [23] and the occurrence of skin rash was related with improved survival with gefitinib for patients with advanced NSCLC [24]. It has been reported that 34 G/A may decrease the *ABCG2* protein expression [25], and thus reduce the transporter activity and increase drug accumulation in the variant *ABCG2*-expressing cells [26]. It may cause a better response to the drug. In addition, there was no significant influence of *ABCG2* 421 C/A on any of the clinical outcome of TKI therapy in our study, which was consistent with a previous study that there was no evident association between *ABCG2* 421 C/A polymorphisms and susceptibility to gefitinib-induced adverse effects in Japanese population [27]. Moreover, Rudin *et al.* have demonstrated that there are no correlation between *ABCG2* 421C/A polymorphism and side-effects in erlotinib-treated patients [28]. It has been reported that *ABCG2* 421 C/A polymorphism can decrease *ABCG2* protein expression, and thus reduce the transporter activity and indicate a better clinical outcome [29-31]. Significantly, *ABCG2* 421 C/A polymorphism has been related with higher accumulation of erlotinib and gefitinib [32]. However, Cusatis *et al.* [14] found that there was a strong association between the *ABCG2* 421 C/A polymorphism and diarrhea in NSCLC patients treated with gefitinib. Regarding to 1143 C/T and −15622 C/T, some researchers found a decreased protein expression related to these two polymorphisms [31], while others found no relation between them [18]. Therefore, the relationships between *ABCG2* polymorphisms and clinical outcome of the targeted therapy are worthy to be further investigated. Taking these results into account, *ABCG2* 34 G/A may be a possible predictor of the clinical outcome of TKIs therapy in Chinese NSCLC patients.

Although the correlation between *ABCG2* gene polymorphisms and outcome and prognosis of TKIs therapy in Chinese NSCLC patients was estimated, some limitations still remain in this study. The number of patients treated with TKI therapy was only 70. Moreover, the TKI-induced side effects (such as diarrhea and skin toxicity) were not considered in this study. Additionally, exon 19 deletion mutation and exon 21 point mutation in EGFR were not described for this patient set. The relation between *ABCG2* SNPs and gefitinib, erlotinib as well as icotinib was not analyzed separately. Therefore, further studies with large patients should be needed to confirm our results, TKIs induced side effects, exon 19 deletion mutation and exon 21 point mutation in EGFR, and relation between *ABCG2* SNPs and gefitinib, erlotinib as well as icotinib should be taken into consideration in further study.

## Conclusions

In conclusion, a strong association between the *ABCG2* 34 G/A polymorphism and the OS of NSCLC patients treated with TKIs (gefitinib, erlotinib and icotinib) indicates that *ABCG2* 34 G/A may be a possible predictor of the clinical outcome of TKIs therapy in Chinese NSCLC patients.

## Additional file

Additional file 1: Table S1. The evaluation of risk factors for overall survival in patients with non-small cell lung cancer treated with TKIs using univariate and multivariate Cox proportional hazards analysis.

## Abbreviations

*ABCG2*: ATP binding cassette superfamily G member 2
CR: Complete response
EDTA: Ethylenediamine tetra-acetic acid
EGFR: Epidermal growth factor receptor
NSCLC: Non-small-cell lung cancer
OS: Overall survival
PD: Progression disease
PFS: Progression-free survival
PR: Partial response
SD: Stable disease
SNPs: Single nucleotide polymorphisms
TKIs: Tyrosine kinase inhibitions
