# Supplementary material for: Impact of ABCG2 polymorphisms on the clinical outcome of TKIs therapy in Chinese advanced non-small-cell lung cancer patients
Source: Cancer Cell Int. 2015 Apr 19;15:43. doi: 10.1186/s12935-015-0191-3 (PMC4425882; doi:10.1186/s12935-015-0191-3)
Supplement: Additional file 1: Table S1. — The evaluation of risk factors for overall survival in patients with non-small cell lung cancer treated with TKIs using univariate and multivariate Cox proportional hazards analysis. [file 12935_2015_191_MOESM1_ESM.doc]

Additional file 1: Table S1. The evaluation of risk factors for overall survival in patients with non-small cell lung cancer treated with TKIs using univariate and multivariate Cox proportional hazards analysis

| Prognosis factors | | univariate analysis | | | multivariate analysis | | |
| --- | --- | --- | --- | --- | --- | --- | --- |
|  |  | P value | HR | 95%CI | Pvalue | HR | 95%CI |
| Clinical outcome  (Sensitive vs Resistive） | | 0.083 | 0.590 | 0.325-1.071 | 0.089 | 0.582 | 0.312-1.085 |
| Gender | Male vs Female | 0.092 | 1.651 | 0.922-2.955 | 0.219 | 1.718 | 0.725-4.074 |
| Age | ≤63 vs>63 | 0.294 | 1.371 | 0.761-2.472 | 0.171 | 1.550 | 0.828-2.903 |
| Smoking | Never vs Ever | 0.227 | 1.477 | 0.785-2.779 | 0.678 | 0.806 | 0.292-2.226 |
| Histology | Others vs Adeno | 0.187 | 2.213 | 0.681-7.194 | 0.158 | 2.459 | 0.706-8.566 |
| G34A | GG vs GA+AA | 0.006 | 1.526 | 1.128-2.065 | 0.004 | 1.765 | 1.193-2.611 |
| C1143T | CC vs CT+TT | 0.873 | 1.026 | 0.748-1.409 | 0.133 | 0.717 | 0.464-1.106 |
| C421A | CC vs CA+AA | 0.824 | 0.967 | 0.718-1.302 | 0.981 | 0.996 | 0.710-1.397 |
